# Joint-Linker Type Ionic Gels Using Polymerizable Ionic Liquid as a Crosslinker via Thiol-Ene Click Reactions

**DOI:** 10.3390/polym12122844

**Published:** 2020-11-29

**Authors:** Kumkum Ahmed, Aoi Inagaki, Naofumi Naga

**Affiliations:** 1 SIT Research Laboratories, College of Engineering, Shibaura Institute of Technology, 3-7-5 Toyosu, Koto-ku, Tokyo 135-8548, Japan; 2 Graduate School of Engineering and Science, Shibaura Institute of Technology, 3-7-5 Toyosu, Koto-ku, Tokyo 135-8548, Japan; mc20005@shibaura-it.ac.jp; 3 Department of Applied Chemistry, College of Engineering, Shibaura Institute of Technology, 3-7-5 Toyosu, Koto-ku, Tokyo 135-8548, Japan

**Keywords:** Ionic gel, ionic liquid, thiol-ene reaction, multifunctional thiols, Li ion

## Abstract

In this work, we report the synthesis of ion-conductive gels, or ionic gels, via thiol-ene click reactions. The novel gel systems consist of the multifunctional thiol monomers tris[(3-mercaptopropionyloxy)-ethyl]-isocyanurate (TEMPIC), pentaerythritol tetrakis(3-mercaptopropionate) (PEMP), and dipentaerythritol hexakis(3-mercaptopionate) (DPMP) as joint molecules and bifunctional allyl ionic liquid (IL) as a crosslinker. The thiol-ene reaction was carried out in lithium bis(trifluoromethanesulfonyl)imide (Li-TFSI) in a propylene carbonate (PC) (1 M) solvent system via a photopolymerization process. The chemical structure and mechanical, thermal, and conductive properties of the gels were investigated using Fourier transform infrared (FTIR) spectroscopy, thermogravimetric analysis (TGA), compression tests, and impedance spectroscopy, respectively. The mechanical and conductive properties of the ionic gels were found to be largely dependent on the monomer content and functionalities of the joint molecules. TGA revealed good thermal stability of the gels up to 100 °C. An ionic conductivity of 4.89 mS cm^−1^ was realized at room temperature (298 K) for low-functional thiol monomers, and a further increase in ionic conductivity was observed with an increase in Li+ ion content and temperature.

## 1. Introduction

Polymeric gels have created well-adjusted fusion in the fields of physics, chemistry, and material science, resulting in significant advancements in the properties of gels and functional materials. A wide variety of polymeric gels, such as physical gels, hydrogels (double network hydrogels, shape memory hydrogels, interpenetrating polymer networking [IPN] hydrogels, etc.), organogels, ionogel/ionic gels, and aerogels have been developed for diverse applications in numerous fields [1,2,3,4,5]. Among them, gels containing ionic groups in their polymeric network offer numerous prospects in the electrochemistry and energy sectors. One of the most promising methods of generating ionic conductivity in gels is incorporating an ionic liquid (IL)-based solvent in the reaction system or choosing a polymerizable IL as a monomeric unit. ILs are salts with poorly coordinated ions, which results in these solvents being liquid below 100 °C or even at room temperature. They offer several attractive properties, such as non-volatility, non-flammability, a wide electrochemical window, high thermal stability, and high ionic conductivity. These characteristics have made them potential candidates for use in gel polymer electrolytes and batteries [6,7,8]. Previously, we developed various types of joint-linker gels with different reaction mechanisms and molecular designs that show the possibility of synthesizing gels with homogeneous, controlled mesh sizes and designed internal network structures [9,10,11,12]. The thiol-ene reaction allows such designable chemistry with significantly robust yet simple, rapid, and facile techniques for polymer chemistry focusing on both fundamental research and practical implementation [13,14,15,16]. Thiol-ene radical reactions have recently been extended to a variety of synthetic processes for basic chemical synthesis and polymeric material modification, fabrication of a wide range of polymeric materials, and new applications, including optical displays, nanoimprinting, holographic diffractive materials, microfluidic devices, high-impact energy-absorbing devices, complex-surface patterns, optical-switching arrays, 3D printing and so on [13,14,15,16].

Utilization of IL in thiol-ene polymerization has proven to be highly effective and feasible in some recent studies; however, an exceptional range of polymeric materials with tailored properties has yet to be developed [17,18,19]. One of the previous works on polymerizable IL-based gels reported following the mechanism of the thiol-ene reaction and the potential of these gels in pH sensing materials and microcontainers has been discussed; however, no clear information on the mechanical properties or the dependence of monomer functionality on the mechanical and conductive properties has been specified [17]. In our previous study, the synthesis and 3D fabrication of a series of ionic gels formed via a thiol-ene reaction in an IL-based solvent system and characterization of their mechanical, conductive, and thermal properties were discussed [20]. In this study, we introduce a novel ion-conducting polymeric structure by a thiol-ene reaction. A diallyl compound with an IL structure is used as a linker molecule, and a multifunctional thiol compound is used as a joint molecule in a solvent system containing Li ions. The multifunctional thiol-containing monomers (tris[(3-mercaptopropionyloxy)-ethyl]-isocyanurate (TEMPIC), pentaerythritol tetrakis(3-mercaptopropionate) (PEMP), and dipentaerythritol hexakis(3-mercaptopionate) (DPMP)) have been chosen to create structural variations in the gel polymers and understand how these structural variants affect the mechanical and conductive behaviors of the gels. The polymerizable IL-based crosslinking unit is incorporated to generate more stable and organized ionic characteristics within the gel network. Finally, a significant increase in ionic conductivity is achieved by using Li-ion containing solvent system. We study the synthesis, as well as the characterization of the physicochemical properties, systematically in order to understand the mechanical, conductive, and thermal behavior of the joint-linker gels.

## 2. Materials and Methods

### 2.1. Materials

Thiol joint molecules, TEMPIC, and PEMP were donated by Showa Denko Co., Ltd, Toyama, Japan. and used without further purification. The joint molecule DPMP was donated by SC Organic Chemical Co., Ltd., Japan and used as received. An IL-based crosslinker, 1,3-diallylimidazolium bis(trifluoromethanesulfonyl) imide (DAIM TFSI), Li salt, and lithium bis(trifluoromethanesulfonyl)imide (Li-TFSI) were purchased from Kanto Kagaku. The photoinitiator benzophenon (BzPh) and solvent propylene carbonate (PC) were purchased from Wako Pure Chemical Industries, Ltd., Japan. Unless mentioned otherwise, all the chemicals were used as received.

### 2.2. Synthesis of Gels

**Figure 1:**
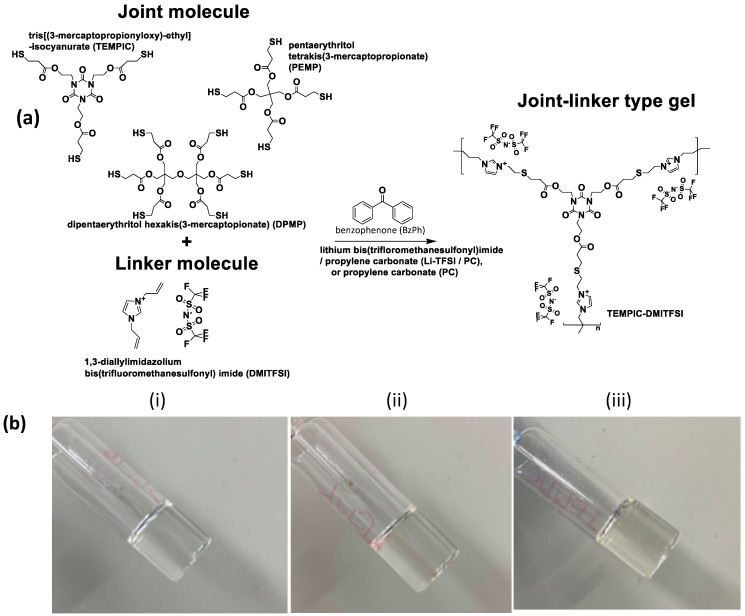
(**a**) Schematic synthesis via thiol-ene reaction between multifunctional thiol monomers and DAIM TFSI linker. (**b**) Images of the gels (**i**) DPMP-DAIM TFSI (**ii**) PEMP-DAIM TFSI and (**iii**) TEMPIC-DAIM TFSI in Li-TFSI/PC (1 M) solvent, monomer concentration: 30 wt %.

Ionic gels were prepared using a one-step photopolymerization process. Desired quantities of thiol joint molecules, IL-based crosslinkers, and Li-TFSI in PC (1 M) were placed in vials and mixed in a vortex mixer, followed by the addition of initiator. The mole ratio of the S–H group in the joint molecule to the allyl group in the crosslinker was adjusted to 1. For preparing gels with 30 wt % total monomeric content (e.g., DPMP joint molecule and DAIM TFSI linker), 0.16 g (0.20 mmol) DPMP, 0.26 g (0.61 mmol) DAIM TFSI, 0.97 g Li-TFSI in PC (1 M), and 5.5 mg (0.030 mmol) BzPh were placed in a vial and mixed thoroughly. The resulting mixture was transferred to an ample tube. Photo-polymerization was carried out using an ultraviolet light-emitting diode (UV LED) source (4000 mW) with a wavelength of 365 nm. UV light irradiation was performed for 10–15 min. The gels without Li-TFSI were prepared following the same procedure as mentioned above in pure PC solvent. Schematic synthesis process and images of the gels are shown in Figure 1.

### 2.3. Method of Characterization

Infrared spectra were acquired using a Jasco FTIR (Fourier transform infrared) 410 Plus Spectrometer in transmission mode over the wavenumber range of 400–3500 cm^−1^. Sample preparation for infrared (IR) measurement was as follows: the pregel solution (containing the monomer, linker, and initiator) was poured between KBr-Real Crystal IR-Card and Slip (International Crystal Laboratories), and the transmission spectra before gelation were collected. Subsequently, this sample was irradiated with UV (4000 mW) light for 30 min in a dark room at room temperature to cause gelation, at which point FTIR measurements were again performed.

Mechanical characterization of the ionic gels was performed using a STA-1150 testing machine (Orientec Co., Ltd., Japan). The sample gels for the compression tests were shaped into a cube with a side length of 10 mm, and the crosshead speed during compression was kept constant at 0.50 mm min^−1^. All tests were carried out at room temperature.

Thermogravimetric analysis (TGA) of the samples were conducted using open aluminum pans on a thermogravimetry/differential thermal analyzer, TG-DTA2020SA, from room temperature to 500 °C at a heating rate of 10 °C min^−1^ under argon atmosphere.

The conductivity of the ionic gels was measured within the frequency range 4 Hz–1 MHz and determined by using a HIOKI 3532–80 chemical impedance analyzer. The ionic gels were prepared in a constant volume cylindrical cell with stainless steel blocking electrodes and a Teflon spacer. Gel samples were sandwiched between mirror-finish stainless-steel electrodes, sealed in a Teflon container, and subjected to impedance measurements. The measurements were taken at controlled temperatures from 20 to 80 °C in an Espec SU-220 temperature-controlled chamber three times. The samples were thermally equilibrated at each temperature for at least 1 h prior to the measurements.

## 3. Results and Discussion

### 3.1. Fourier Transform Infrared (FTIR) Spectroscopy Analysis

**Figure 2:**
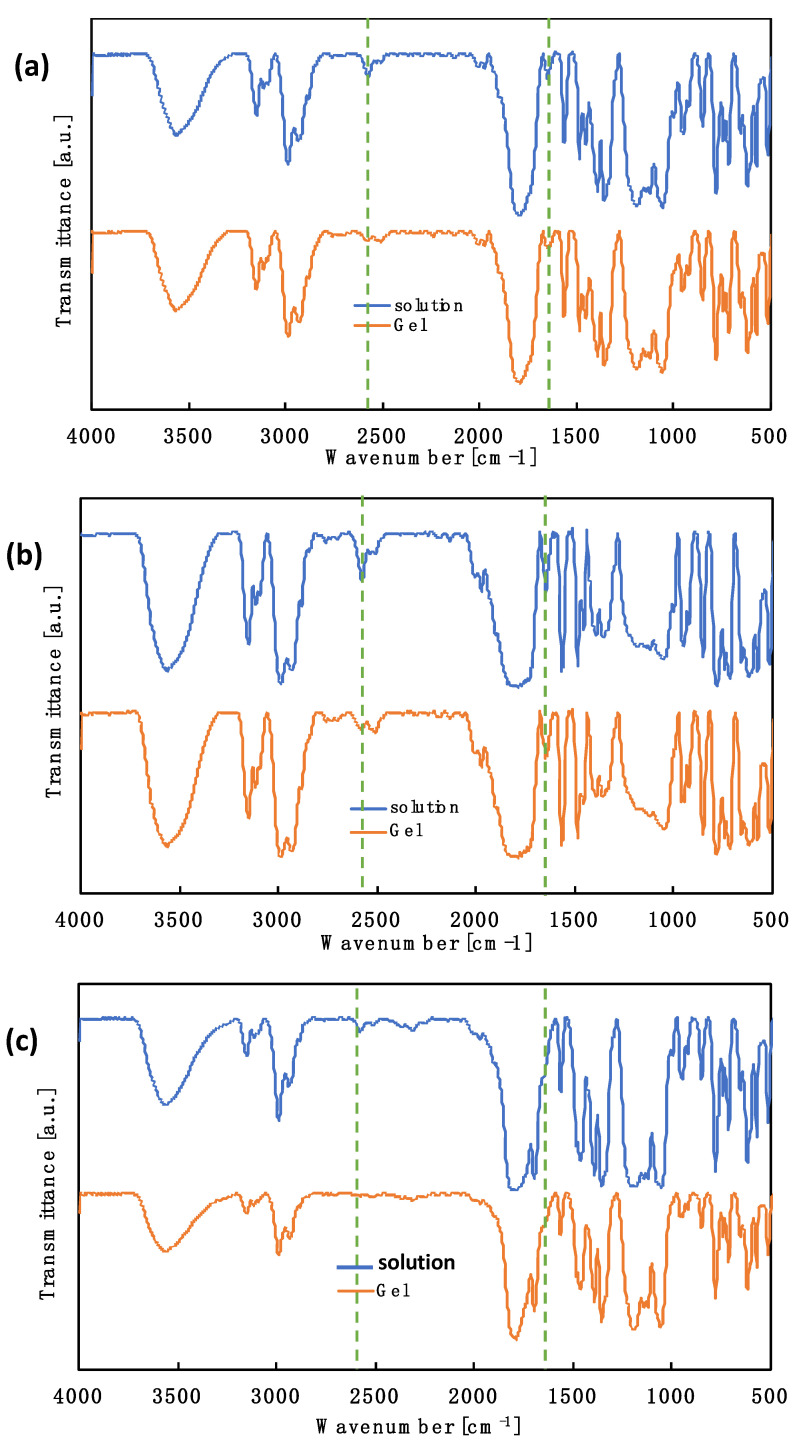
FTIR spectra of (**a**) the DPMP- DAIM TFSI system, before reaction (blue line), after reaction (orange line), solvent: Li-TFSI/PC (1 M), monomer concentration: 30 wt %, (**b**) the PEMP-DAIM TFSI system, before reaction (blue line), after reaction (orange line), solvent: Li-TFSI/PC (1 M), monomer concentration: 30 wt % and (**c**) the TEMPIC- DAIM TFSI system, before reaction (blue line), after reaction (orange line), solvent: Li-TFSI/PC (1 M), monomer concentration: 30 wt %.

Figure 2a–c shows the FTIR spectra of the pregel solution (before reaction) and the gel (after reaction) in each reaction system (i.e., the DPMP-DAIM TFSI system, PEMP-DAIM TFSI system, and TEMPIC-DAIM TFSI system) with a monomer concentration of 30 wt %. In all these systems, the peaks derived from the characteristic peak of the C=C double bond stretching vibration at 1640 cm^−1^ and the stretching vibration of the thiol group (–SH) near 2560 cm^−1^ decreased after the reaction. Thus, the progress of the thiol-ene reaction was confirmed. It can be mentioned here that the peaks responsible for the C=C and –SH groups did not disappear completely, which signifies that there were a few unreacted monomeric and crosslinking units remaining in the gel system. Bands in the region of 2924–2987 cm^−1^ and 3110–3150 cm^−1^ represent the stretching of −CH groups with a peak at 1564 cm^−1^ for the C=C double bonds of the imidazole ring of the IL, which is in good agreement with previously reported ILs containing TFSI anions and imidazolium cations [21,22,23,24].

### 3.2. Mechanical Properties

**Figure 3:**
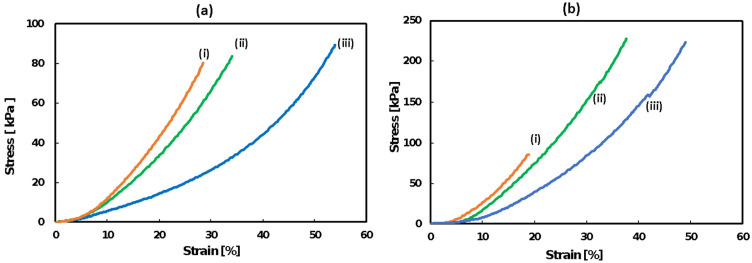
Stress–strain curves of (**a**) gels containing 20 wt % monomer, (i) DPMP- DAIM TFSI, (ii) PEMP-DAIM TFSI and (iii) TEMPIC- DAIM TFSI gels, and (**b**) gels containing 30 wt % monomer, (i) DPMP-DAIM TFSI, (ii) PEMP- DAIM TFSI and (iii) TEMPIC-DAIM TFSI gels; solvent: Li-TFSI/PC (1 M).

Figure 3a,b shows representative stress–strain curves of gels synthesized in each reaction system with monomer concentrations of 20 and 30 wt %. Table 1 gives quantitative information on the mechanical properties, such as Young’s modulus, breaking strain, and breaking stress.

Among all the gels with monomer concentrations of 20 and 30 wt %, DPMP-DAIM TFSI (20 and 30 wt %) showed the highest rigidity, and TEMPIC-DAIM TFSI showed the lowest rigidity, which can be well justified by the fact that the number of crosslinking points in the DPMP monomer is higher due to the higher functionalities. The number of functional groups in each monomer is as follows: DPMP (6 functional) > PEMP (4 functional) > TEMPIC (3 functional). As the monomer functionality decreases, the number of crosslinking points decreases, and consequently, the gels become less rigid and more flexible. In addition, gels with 30 wt % monomer content showed higher rigidity than gels with 20 wt % monomer content, as the crosslinking density increases at higher monomer concentrations.

### 3.3. Thermal Properties

**Figure 4:**
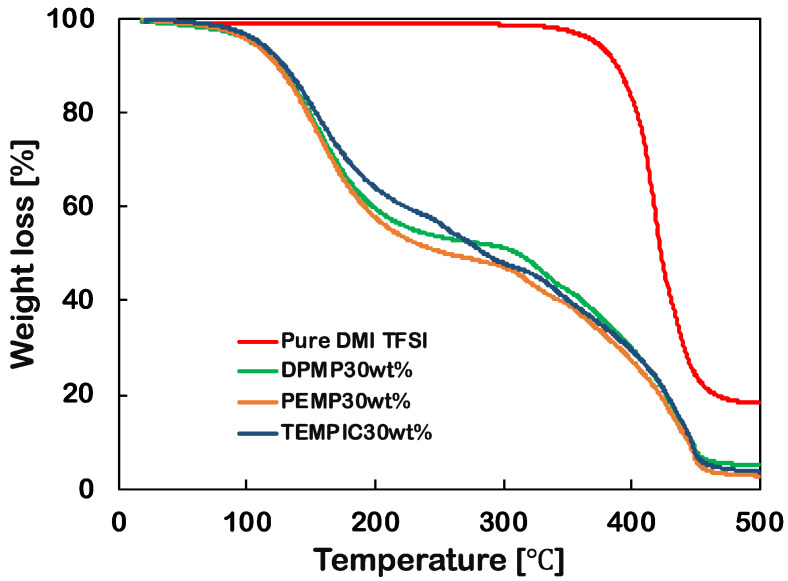
TGA thermograms of DPMP-DAIM TFSI, PEMP-DAIM TFSI, TEMPIC-DAIM TFSI ionic gels and pure DAIM TFSI, solvent: Li-TFSI/PC (1 M), monomer concentration: 30 wt %.

The thermogravimetric properties of the IL-based linker, DAIM TFSI, and ionic gels containing 30 wt % monomer content were measured, and the results are shown in Figure 4 and Table 2. DAIM TFSI exhibits one-step decomposition, while the ionic gels DPMP-DAIM TFSI, PEMP-DAIM TFSI, and TEMPIC-DAIM TFSI show a two-step decomposition pattern. ILs are well known for their high thermal stability. DAIM TFSI also displayed high thermal stability, with decomposition of 5 wt % occurring at temperatures above ~370 °C. For the gel samples, the first decomposition step occurred at approximately 100 °C due to the decomposition of PC, and the second decomposition step, at temperatures greater than 200 °C, was responsible for the degradation of the crosslinked polymer content. The temperature at which the weight loss reached 50 wt % (T_d1/2_) was highest for the DPMP-DAIM TFSI gel, followed by TEMPIC- DAIM TFSI and PEMP-DAIM TFSI gel systems. The reason for the highest T_d1/2_ of DPMP-DAIM TFSI gel might be due to the increased functionalities of DPMP monomers, which lead to an increase in the concentration of crosslinking points in the gel and thus make it more difficult for the gel to decompose. Due to the higher molecular weight of TEMPIC, TEMPIC-DAIM TFSI gel showed slightly higher T_d1/2_ compared to PEMP-DAIM TFSI gel. All the gel systems showed similar degradation profile. The residual mass of all the gels was less than 5 wt %, while for DAIM TFSI, the residual mass was ~18%.

### 3.4. Conductive Properties

**Figure 5:**
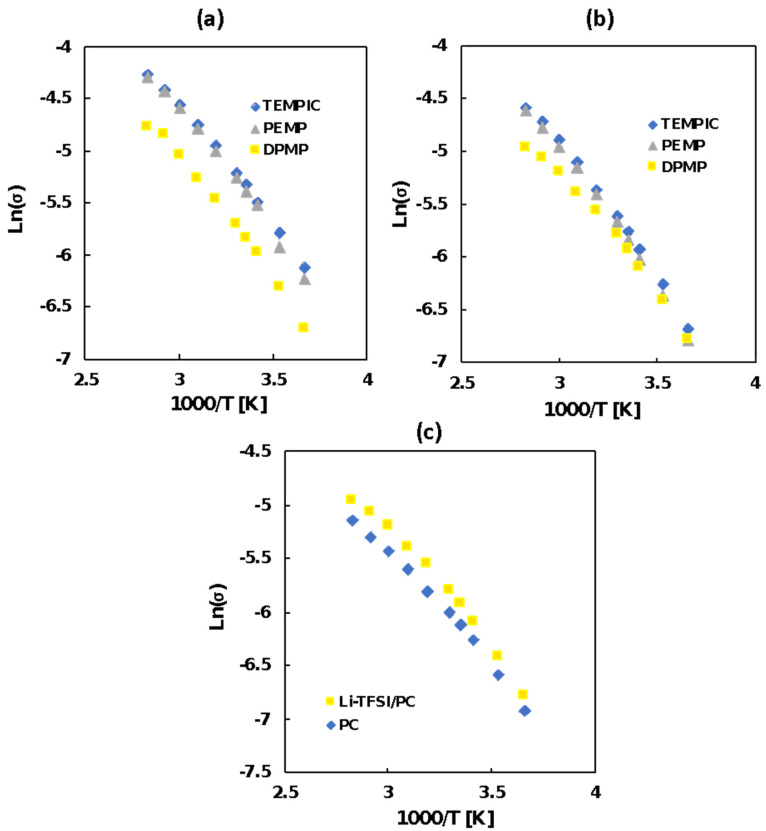
Arrhenius plots of (**a**) 20 wt % and (**b**) 30 wt % monomer-containing DPMP-DAIM TFSI, PEMP-DAIM TFSI, TEMPIC-DAIM TFSI ionic gels in Li-TFSI/PC (1 M) and (**c**) Arrhenius plots of 30 wt % monomer-containing DPMP-DAIM TFSI ionic gels in Li-TFSI/PC (1 M) and PC.

The ionic conductivities of the ionic gels were measured using impedance spectroscopy in the temperature range of 0–80 °C. Figure 5a–c shows the temperature dependent conductivity of the TEMPIC-DAIM TFSI, PEMP-DAIM TFSI and DPMP-DAIM TFSI gels in Li in PC (1 M). The ionic conductivity was highest for the TEMPIC-DAIM TFSI gels and lowest for the DPMP-DAIM TFSI gels at monomer concentrations of 20 and 30 wt %. This behavior is thought to be dependent on the functionalities of the joint molecules. It was observed that the smaller the number of functionalities, the smaller the network structure of the gel, such that the lithium ions added by the solvent are likely to move freely. In addition, the gels containing 20 wt % monomer content had higher ionic conductivity than gels with a monomer content of 30 wt %. This might have occurred due to the increase of the Li+ content as the monomer concentration decreased. Furthermore, the Vogel–Tammann–Fulcher (VTF) parameters in Table 3 show that the higher the ionic conductivity, the higher the number of ion carriers and the lower the activation energy for ion transport.

As described above, the smaller the number of functional groups, the smaller the network structure of the gel and thus the less energy required for ion transport. Regarding the lithium ion diffusion coefficient of the gels, the values for the 20 wt % samples were higher than those for the samples with a monomer concentration of 30 wt % because, at 20 wt % monomer concentration, the content of lithium ions increased, and the number of diffusing ions also increased. Furthermore, the data represented in Figure 5 and Table 3 also show that the gel containing lithium ions in PC showed higher ionic conductivity than the gel containing no lithium ions, i.e., pure PC. Thus, it can be said that both the IL as a linker molecule and the Li ions in solvent contributed to the high ionic conductivity of the gels. In this work, the gels were prepared by choosing the monomers and crosslinkers in a stoichiometric ratio so that a well uniformed network structure following joint-linker mechanism is formed. In a non-stoichiometric system where crosslinker (diallylic IL) is used in a larger proportion than the monomers (thiol compound), it might be possible to increase the conductivity of the gels thus offering a possible way to further tune the mechanical and thermal properties of the ionic gels. However, for a non-stoichiometric system, non-uniform network structures with unreacted vinyl/thiol groups are expected to form. Such a phenomenon will be studied in the near future.

## 4. Conclusions

We successfully fabricated a novel type of multifunctional ionic gel with variable structures and properties via thiol-ene click chemistry. The mechanical properties were found to be significantly dependent on the total monomer concentration and functionality of the joint molecules. Highly functional joint molecules exhibited enhanced mechanical rigidity. Among the three types of joint molecules, the low functional type (TEMPIC) showed a more ductile nature than the gels with highly functional joint molecules (PTMB, DPMP) due to the enhanced crosslinking by highly functional thiol groups. A thermal stability study by TGA exhibited a favorable degradation temperature up to 100 °C. Incorporation of IL in the monomeric unit and Li ion in the solvent has been found to be advantageous for gels, as all the gels exhibited high ionic conductivity. Due to their flexible nature, derived from a low crosslinking density, gels with low functional joint molecules (TEMPIC) exhibited the highest ionic conductivity. Thus, it can be said that these gels with tunable mechanical properties, conductivity, and favorable thermal stability are very suitable for applications in lithium-ion batteries, sensors, and other electrochemical devices.

## Figures and Tables

**Table 1:** Mechanical properties of joint-linker type gels.

Joint Molecule	Monomer Concentration [wt%]	Young’s Modulus [kPa]	Breaking Strain [%]	Breaking Stress [kPa]
DPMP	20	352.4	29.2	80.3
PEMP	20	268.8	30.7	84.0
TEMPIC	20	71.0	33.8	89.2
DPMP	30	459.0	17.4	96.8
PEMP	30	387.1	32.5	227.8
TEMPIC	30	190.8	44.5	224.2

**Table 2:** Thermal properties of ionic gels by TGA.

Sample	T_d_ [°C]		
	5 wt %	50 wt %	Remaining
Pure DAIM TFSI	372.4	422.9	475.7
DPMP-DAIM TFSI	103.8	311.7	465.3
PEMP-DAIM TFSI	104.3	256.6	465.1
TEMPIC-DAIM TFSI	109.5	285.5	465.5

**Table 3:** Vogel–Tammann–Fulcher (VTF) equation parameters and apparent lithium ion diffusion coefficients (Dapp) for ionic gels.

Joint Molecule	Monomer Concentration [wt %]	A [S cm^−1^]	B [K]	T_0_ [K]	D_app_ [10^−14^ cm^2^ s^−1^]	Conductivity at 298 K [mS cm^−1^]
TEMPIC	20	3.8	468.1	175.9	1.8	4.89
PEMP	20	3.6	472.3	174.2	1.8	4.60
DPMP	20	2.7	530.2	165.3	1.7	2.91
TEMPIC	30	3.1	488.7	176.5	1.2	3.14
PEMP	30	3.1	494.3	177.5	1.2	2.91
DPMP	30	2.2	554.6	155.3	1.3	2.77
DPMP/PC	30	1.8	562.0	153.5	-	2.22
